# Bioactive PI3-kinase/Akt/mTOR Inhibitors in Targeted Lung Cancer Therapy

**DOI:** 10.34172/apb.2023.003

**Published:** 2021-10-09

**Authors:** Somayyeh Ghareghomi, Vahideh Atabaki, Naseh Abdollahzadeh, Shahin Ahmadian, Salar Hafez Ghoran

**Affiliations:** ^1^Department of Biochemistry, Institute of Biochemistry and Biophysics (IBB), University of Tehran, Tehran, Iran.; ^2^Department of Pharmacognosy and Pharmaceutical Biotechnology, Faculty of Pharmacy, Hormozgan University of Medical Sciences, Bandar Abbas, Iran.; ^3^Neurophysiology Research Center, Cellular and Molecular Medicine Institute, Urmia University of Medical Sciences, Urmia, Iran.; ^4^Phytochemistry Research Center, Shahid Beheshti University of Medical Sciences, Tehran, Iran.; ^5^Medicinal Plants Research Center, Yasuj University of Medical Sciences, Yasuj, Iran.

**Keywords:** Alkaloids, Cancer treatment, Drug discovery, Flavonoids, Lung cancer, PI3K/Akt/mTOR

## Abstract

One of the central signaling pathways with a regulatory effect on cell proliferation and survival is Akt/mTOR. In many human cancer types, for instance, lung cancer, the overexpression of Akt/mTOR has been reported. For this reason, either targeting cancer cells by synthetic or natural products affecting the Akt/mTOR pathway down-regulation is a useful strategy in cancer therapy. Direct inhibition of the signaling pathway or modulation of each related molecule could have significant feedback on the growth and proliferation of cancer cells. A variety of secondary metabolites has been identified to directly inhibit the AKT/mTOR signaling, which is important in the field of drug discovery. Naturally occurring nitrogenous and phenolic compounds can emerge as two pivotal classes of natural products possessing anticancer abilities. Herein, we have summarized the alkaloids and flavonoids for lung cancer treatment together with all the possible mechanisms of action relying on the Akt/mTOR pathway down-regulation. This review suggested that in search of new drugs, phytochemicals could be considered as promising scaffolds to be developed into efficient drugs for the treatment of cancer. In this review, the terms "Akt/mTOR", "Alkaloid", "flavonoid", and "lung cancer" were searched without any limitation in search criteria in Scopus, PubMed, Web of Science, and Google scholar engines.

## Introduction

 Lung cancer is now the most commonly identified solid tumor and the most common cause of cancer death worldwide. It is estimated that there are 1.59 million deaths from lung cancer every year.^1^ Despite the use of various strategies for treating lung cancer, these strategies are not satisfactory and the recovery of these patients is not as expected.^2,3^ There are two main forms of lung cancer: small-cell lung cancer (SCLC; about 15% of all lung cancer) and non-small-cell lung cancer (NSCLC; about 85% of all lung cancer). Despite developments in standard treatment and early detection, NSCLC has a weak prognosis and is mostly diagnosed at an advanced stage. This kind of lung cancer includes three major histologic subtypes: large-cell, adenocarcinoma, and squamous cell carcinoma.^4,5^ Protein kinase B (PKB), or Akt, is a serine/threonine protein kinase. Akt molecule as an essential effector has a critical role in specific cellular processes such as metabolism, proliferation, regulating cell growth, and survival.^6^ Activated Akt agitates various processes including translation of proteins and their modification via activating its downstream proteins.^7^ Reducing cell cycle inhibitors by activated Akt increases cell processes and cell cycle activity. This can reduce apoptosis by inactivating cell death protease and suppressing pro-apoptotic proteins.^8,9^ Activated Akt is one of the important kinases that contribute to various cancers as well as lung cancer. Lung cancer treatment through various strategies and compounds has been investigated in different studies. Plants have provided phytochemicals or secondary metabolites that are increasingly used against different cancers. Among the secondary compounds, flavonoids and alkaloids are of interest in terms of therapeutic activities. Flavonoids are polyphenolic compounds found in plant-based food and many medicinal plants. They are divided into several subclasses including anthocyanins, flavonols, flavan-3-ols, flavones, flavanones, proanthocyanidins, and isoflavones.^10^ Flavonoids indicated potent anti-cancer activity against various cancer models *in vitro* and *in vivo*, mediated through regulating key signaling pathways involved in the migration and invasion of cancer cells and the metastatic process.^11^ They also play a key role in preventing lung cancer. Two clinical trials were undertaken on the patients with lung cancer who received flavonoids epigallocatechin gallate and polyphenon E and, treatment with these compounds resulted in decreased radiation therapy oncology group (RTOG) scores and pain scores compared to the baseline.^12^ Alkaloids represent a diverse chemical group with at least one basic nitrogen atom in their structures. They are specifically distributed in higher plants. These alkaloids are used for drug discovery and most of them show anti-proliferative and anticancer effects on various cancers, both* in vivo* and* in vitro*.^13,14^ Alkaloids may promote cytostatics in drug-resistant cancer cells, induce cell cycle arrest, and be involved in inhibiting tumor metastasis and proliferation.^15^ Some of them, such as camptothecin and vinblastine, have been used as chemotherapeutic agents. Moreover, the National Cancer Institute of Canada JBR.10 trial showed that a *Vinca* alkaloid, vinorelbine plus cisplatin, improved overall survival for stage IB–II NSCLC. Cisplatin is a chemotherapeutic drug commonly used for the first-line treatment of cancers including lung, bladder, ovarian, testicular, etc.^16^ One of the most attributed mechanisms to alkaloids and flavonoids is their ability in the down-regulation of the Akt/mTOR signaling pathway in cancer cells. Although there are review articles aimed at underacting the role of this pathway in cancer, there has been no comprehensive review on the secondary metabolites that affect this target in a specific type of cancer up to now. In this review, therefore, we pointed out the inhibitors of the Akt/mTOR signaling pathway (by focusing on alkaloids and flavonoids) in different lung cancer cell lines in detail. Moreover, the mentioned bioactive compounds were structurally classified here, which that can give insight to drug discovery researchers to find out chemical scaffolds with the Akt/mTOR inhibitory property.

## Akt/mTOR signaling pathway and its role in lung cancer cells

**Figure 1:**
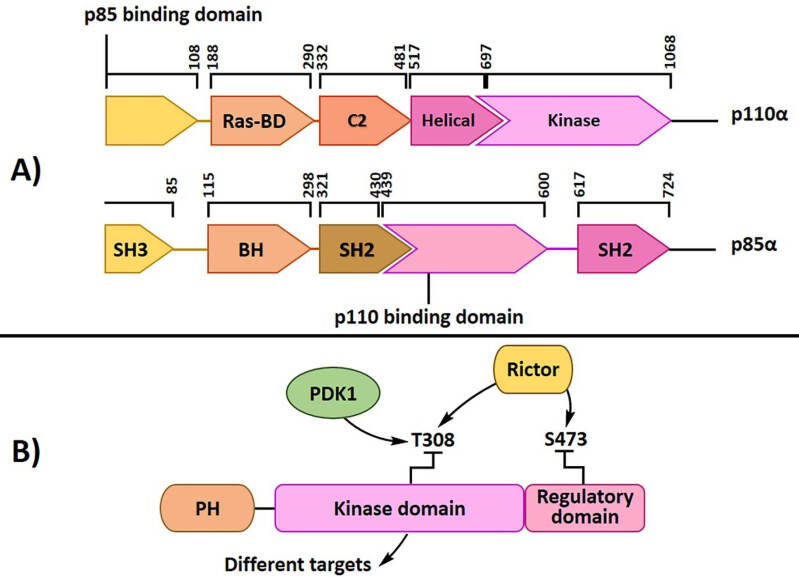
PI3K subunits and domains (A) and Akt domains (B)

 Phosphatidylinositol 3-kinase (PI3-K) is a heterodimer enzyme with p110 catalytic and p85 regulatory subunits (Figure 1A). There are two types of this enzyme (IA and IB), which are different in their subunits. Receptor tyrosine kinase activates class I PI3K with the association of its through one or two SH2 domains in the adaptor unit binding to phosphotyrosine consensus motifs. PI3-K catalyzes the phosphorylation of lipid substrate phosphatidylinositols, such as PI (4,5), P2, and PI (4) P, and the conversion into PI-3,4,5-P3 (PI3P), which activates downstream signaling pathways by the phosphorylation of various kinases such as PDK and Akt.^17^ PKB, or Akt, is a serine/threonine-protein kinase that regulates cell survival, proliferation, growth, apoptosis, and glycogen metabolism.^18,19^ Akt has a short carboxyl-terminal regulatory domain (RT), a crucial catalytic domain, and an amino-terminal Pleckstrin-homology (PH) domain (Figure 1B).^2,20^ Activated PI3K recruits Akt through direct interaction with its PH domain; then, another PH domain-containing 3-phosphoinositide-dependent protein kinase (PDK), serine/threonine kinase, phosphorylates Akt on serine 473 (S473) and threonine 308 (T308) and causes AKT activation.^21,22^ Afterwards, Akt can phosphorylate a variety of downstream protein substrates, including Bcl-2-associated death promoter (BAD), glycogen synthase kinase-3β (GSK-3β), forkhead in rhabdomyosarcoma, and mouse double minute 2 homolog (Mdm2).^23^ Activated Akt stimulates protein translation and cell cycle processing; it also reduces apoptosis by suppressing pro-apoptotic proteins.^8^ According to various studies, Akt is one of the most hyperactive kinases in different human cancers such as lung cancer. Akt phosphorylation was detected in human bronchial epithelial cells before malignancy^24^; activated Akt was also discovered in pre-neoplastic bronchial lesions and an increase in the incidence and progression of lung cancer.^25^ Phosphorylation and hyperactivation of Akt were detected in 30-75% of NSCLCs.^26^ Furthermore, phospho-Akt (p-Akt) was shown in 70% of SCLCs patients by immune-histochemical analysis,^27^ which proved the role of Akt hyperactivation in lung cancer progression. Various hereditary and environmental factors are involved in developing lung cancer. Different studies have shown that Akt has an essential role in lung cancer cells. Malanga et al reported that Akt1 hyperactivity due to E17K point mutation might cause significant progression in these cancers.^28^ Smoking is the leading cause of lung cancer and 85-90% of patients use tobacco.^29^ Tobacco carcinogen prompts PI3K-dependent activation of Akt in the lung epithelial cells.^30^ Moreover, tobacco constituents can activate the PI3K/Akt pathway by activating various upstream signals of PI3K, containing Ras, growth factor tyrosine kinase receptor, and phosphatase tensin homolog deleted on chromosome ten (PTEN). Epidermal growth factor receptor (EGFR) is a main upstream signal of PI3K. Overexpression of EGFR has a close interaction with tobacco use and was detected in bronchial epithelial cells of smokers.^31^ Over-expression of EGFR is also specified in 40-80% of patients with NSCLCs. Some studies have shown that some mutations in EGFR can lead to its overexpression^32^ such as point mutation in exon 18, insertion in exon 20, deletion in exon 19, and point mutation in exon 21,^33^ among which the last two are very important. According to various studies, there is an important linkage between PI3K/Akt and Ras signaling.^34^ K-ras mutation is responsible for 25% of smoking-associated human lung adenocarcinomas and boosts motility and invasiveness of lung adenocarcinoma cells through Akt activation.^35^ PTEN is a lipid phosphatase that can negatively regulate the PI3K/Akt pathway via PIP3 dephosphorylation at the plasma membrane.^36^ When this phosphatase is mutated, it cannot convert PI3P into PIP and the PI3K/Akt signaling pathway will be hyperactive. PTEN mutations are involved in almost 70% of NSCLCs through inactivating mutations^37^ and cause constitutively activated the PI3K/Akt signaling pathway. Overexpression of various microRNAs, such as miR-21, miR-221, and miR-222,^38,39^ as well as methylation of PTEN promoter and homogenous deletion of PTEN gene, are the most significant mechanisms for reducing PTEN activity in lung cancer.^40^ Therefore, different pathways correlate with the hyperactivation of Akt signaling and the growth and progression of lung cancer cells. Regulating various cellular processes plays an important role in lung tumorigenesis by Akt hyperactivation. One of the most essential downstream proteins associated with Akt is the mammalian target of rapamycin (mTOR). The interaction of Akt and mTOR stimulates the growth and survival of cancer cells.^41^ Activation of the Akt/mTOR signaling pathway induces tumorigenesis via regulating cell growth and progression, protein synthesis, and metabolism. Hyperactivation of Akt/mTOR was shown in 74% of NSCLCs.^41^ mTOR is composed of two separate complexes, mTOR complex 1 and 2 (mTORC1 and mTORC2).^42^ Akt activation by various factors is due to the inhibition of tuberous sclerosis complex (TSC 1/2) and activation of mTORC1. By the inactivation of eIF4E binding protein and activation of p70 ribosomal protein S6 kinase (S6K1), mTORC1 can then increase ribosome biogenesis and various protein synthesis that are essential for proliferation, cell cycle growth, angiogenesis, and survival pathways.^42^ Besides, mTORC2 phosphorylated protein kinase B (Akt) at serine-473 can contribute to cell growth and progression.^43^ One of the utmost mechanisms for cell cycle progression is the increase in cell cycle promoter cyclin D1 and the inactivation of cell cycle inhibitors, p27 and p21. Cyclin D1 is involved in the transition of G1 to S in the cell cycle. Overexpression of cyclin D1 has been shown in some studies and it has a close correlation with NSCLCs tumorigenesis from the early stage, hence, it can be a molecular biomarker in cancer.^44^ Activation of the Akt signaling pathway has been shown to increase cell survival through inhibiting some pro-apoptotic proteins, such as BAD, and increasing various anti-apoptotic factors such as survival.^45^ On the other hand, Akt can inhibit apoptosis by influencing FOXO protein. Activated FOXO proteins are transferred to the cytoplasm from the nucleus and separate from their pro-apoptotic gene targets.^46^ Activated Akt can also induce FOXO protein degradation through its phosphorylation.^47^ Based on previous studies, neo-angiogenesis in lung cancer has a close correlation with activated Akt. Hypoxia is an important factor for solid tumors enhancing the hypoxia-inducible factor-1 (HIF-1).^48-50^ HIF-1 can increase vascular endothelial growth factor (VEGF) expression that is an essential component of endothelial vascular permeability and cell proliferation.^51^ One of the leading causes of cancer mortality is metastasis that occurs at different rates in various cancers.^52^ Numerous factors are involved in the development and metastasis. Matrix metalloproteinases (MMPs) show a specific function in cancer metastasis.^53^ Several types of MMPs, such as MMP-2, MMP-7, and MMP-9, increase in patients with lung cancer.^54,55^ In some studies, it is observed that Akt signaling activation has the main link to MMPs expression and its regulation.^55^ Accordingly, the Akt/mTOR pathway targeting lung cancer cells can have useful effects on growth control and proliferation. Herbal compounds with the least side effects and maximum impression on cancer cell progression have been observed by various studies. This review summarizes two main groups of natural compounds, covering diverse structural alkaloids and flavonoids with anticancer properties and their possible mechanisms are reviewed relying on the down-regulation of the Akt/mTOR pathway in lung cancer cells.

## Alkaloids and flavonoids as biologically effective metabolites in lung cancer treatment

**Figure 2:**
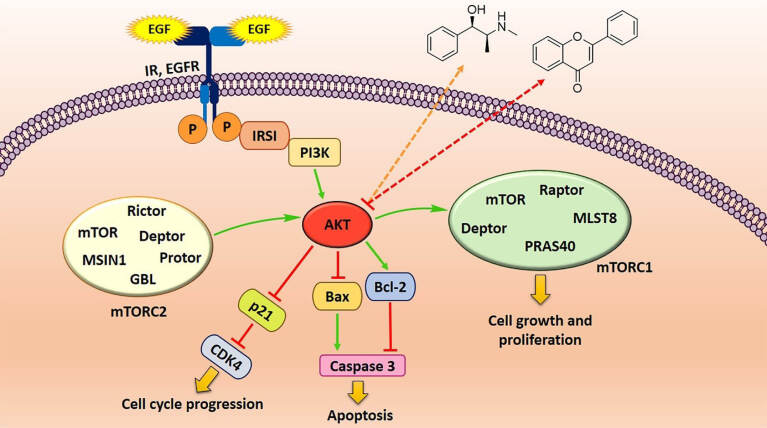
Down-regulation of Akt/mTOR signaling pathway in lung cancer cells by alkaloids and flavonoids

 Natural products have a long history in cancer therapy. Several clinically applied phytochemicals or their semisynthetic derivatives include vincristine, vinblastine, etoposide, teniposide, and taxol.^56^ Flavonoid compounds belong to the polyphenol class of natural products consisting of two aromatic rings (A & B) and an oxygenated heterocyclic ring (C). Depending on the structure of the C ring, they are classified as flavonols, flavones, catechin/condensed tannins, antocyanidine, and isoflavones.^57^ Flavonoids have been investigated to be potential compounds with a broad spectrum of pharmacological effects including their capacities as anticancer agents. Ligand-receptor interaction and antioxidants activities are considered as the main properties of flavonoids as beneficial agents in health.^58^ Cancer chemoprevention of flavonoids in many types of cancer, such as lung cancer, arises from their various features including anti-oxidant, anti-proliferation, anti-angiogenesis, anti-metastasis, and immunoregulatory activities.^59^ Alkaloids are a different group of natural compounds with a cyclic structure containing at least one nitrogen atom. They are distributed in many of the plants belonging to Ranunculaceae, Leguminosae, Loganiaceae, Menispermaceae, and Papaveraceae families.^13^ A wide variety of biological activities, including anti-malarial, anti-asthmatic, anti-cancer, vasodilatory, anti-arrhythmic, analgesic, anti-bacterial, and anti-hyperglycemic properties, have been reported for alkaloids. Many of alkaloids have been tested for their anticancer activity and some have even been approved by FDA for cancer therapy. Several mechanisms, including DNA cleavage mediated by the inhibition of topoisomerase I & II, induction of mitochondrial permeabilization, mitotic arrest, and inhibition of enzymes, are involved in cell signaling.^60^ One of the important mechanisms for the function of these compounds in cancer cells is the down-regulation of the PI3K/AKT/mTOR signaling pathway (Figure 2). Therefore, examining their molecular aspects could open a new perspective on the use of these compounds. In the following, we introduce various kinds of natural subclasses belonging to the alkaloid and flavonoid backbones.

###  Alkaloids regulate the PI3K/Akt/mTOR pathway in lung cancer 

**Figure 3:**
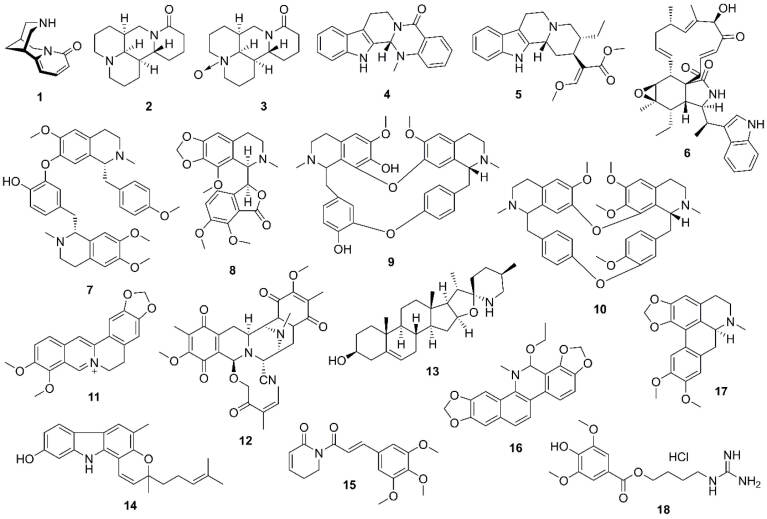
Chemical structures of natural alkaloids (1-18)

 To have a comprehensive literature review, the anti-cancer natural alkaloids targeting the PI3K/Akt/mTOR signaling pathway in lung cancer are categorized according to their chemical classes (Figure 3) while the mechanisms of actions are also summarized for each class of alkaloids where available (Table S1; Supplementary file 1).

####  Quinolizidine alkaloids

 The roots of *Papilionaceae* and *Caesalpinioideae*familiesare used in traditional Chinese medicine, which contains a quinolizidine alkaloid, cytisine (**1**) (Figure 3). To study the anti-tumor effects of compound **1** on lung cancer, Xu et al found that **1** caused cell cycle arrest and apoptosis in A549 cells mediated through ROS generation via MAPK/STAT/NF-кB and Akt signaling pathways, respectively.^61^ Matrine (**2**) (Figure 3) isolated from the Chinese medicinal plant, *Sophora flavescens* Aiton (Fabaceae), is responsible for the induction of apoptosis in 95D and A549 cells via Akt inactivation. The total and phosphorylated Akt expression levels were down-regulated by treatment with 2 in a dose-dependent manner. Compound **2** also promoted apoptosis via reducing the expression of c-IAP_1_ and c-IAP_2_, the anti-apoptotic proteins.^62^ Another potential compound found in *Sophora *species was oxymatrine (**3**) (Figure 3), which exhibited G0/G1 cell cycle arrest in HCC827 cells by inhibiting EGFR as a tumorigenesis agent in lung cancer cells. Interestingly, Akt inhibition partially required oxymatrine-mediated apoptosis.^63^

####  Indole alkaloids

 The sonic hedgehog/GLI family zinc finger 1 (SHH/GLI1) signaling pathway is an oncogenesis pathway activated in lung cancer. Evodiamine (**4**) (Figure 3), an indole alkaloid obtained from *Evodia rutaecarpa* (Juss.) Benth. (Rutaceae),not only inhibited A549 cell proliferation by decreasing the SHH/GLI1 signaling pathway but also blocked the inhibition of the Akt/NF-кB signaling pathway.^64,65^ Hirsutine (**5**) (Figure 3), extracted from *Uncaria rhyncophylla* (Miq.) Jacks (Rubiaceae), is believed to show anticancer activity in lung cancer cell lines via the mitochondrial apoptosis process. Furthermore, compound **5** interrupted the ROCK1/PTEN/P13K/Akt signaling pathway and consequently led to GSK-3β-mediated mitochondria apoptosis when examined in A549 cells and A549 xenograft mouse model.^66^ Chaetoglobosin K (**6**) (Figure 3) is a cytochalasin-derived alkaloid isolated from *Diplodia macrospora* Earle. It has been reported that **6** inhibits Akt and JNK pathways in H1299 and H2009 cells. Compound **6** also reduces the phosphorylation of Akt (ser473 active site) together with JNK (thr183/tyr185 active site) without any alteration in total Akt kinase and total JNK levels.^67^

####  Isoquinoline alkaloids

 Neferine (**7**) (Figure 3) is a major bisbenzylisoquinoline alkaloid of *Nelumbo nucifera *Gaertn. (also known as Lotus; Nelumbonaceae). Compound **7** induced autophagy in human lung adenocarcinoma cells (A549) through the following two pathways, P13K/Akt/mTOR and ROS hypergeneration. It also caused the acidic vesicular organelle formation and conversion of autophagosome markers, LC3B-І and LC3B-II, which are involved in the canonical form of autophagy. Neferine-induced non-canonical autophagy was conducted through the down-regulation of P13K, Akt, and mTOR, in addition to inhibition of P13K/Akt/mTOR pathway.^68^ However, the expression of P13K/Akt/mTOR genes was down-regulated by **7** treatment *in vivo* diethylnitrosamine-induced lung carcinogenesis.^69^ Moreover, **7** could increase cisplatin-induced autophagy in A549. Significant pieces of evidence were observed when a combination of neferine and cisplatin down-regulated the expression of protein and mRNA of the P13K/Akt/mTOR pathway. These effects not only significantly reduced the phosphorylation of amino acids, Ser473, and Thr308 in Akt protein but also decreased the expression of p85 subunit of P13K protein and mTOR levels.^70^ Liu et al showed that co-treatment with neferine and ethoxysanguinarine, enhanced respectively cisplatin-induced autophagy and apoptosis in lung cancer cells, associated with down-regulation of PI3K/AKT/mTOR pathway.^71^ It has been shown that the synergistic effect of cisplatin and noscapine (**8**) (Figure 3), an opium alkaloid from *Papaver somniferum*L., the Papaveraceae family, resulted in decreased the Akt expression level, survived proteins, and increased the expression of proapoptotic proteins in H460 cells.^72^ Mutations of proto-oncogene KRAS are found in 20-30% of patients with NSCLC that can activate downstream pathways, most significantly the RAS-P13K-Akt and RAS-RAF-ERK. Krukovine (**9**) (Figure 3), which is a bisbenzylisoquinoline alkaloid of *Abuta grandifolia* (C.Mart.) Sandwith (Menispermaceae),^15^ effectively targeted the downstream pathways in A549 and H460 cell lines with KRAS mutations. Compound **9** down-regulated RAF-ERK and inhibited the Akt phosphorylation pathway resulting in inducing the G1 apoptosis and arrest in KRAS-mutated lung cancer cells.^73^ Tetrandrine (**10**) (Figure 3), a bis-benzylisoquinoline component yielded from *Stephania tetrandra* S. Moore (Menispermaceae), induced apoptosis and inhibited proliferation in A549 cells by the down-regulation of ERK and Akt phosphorylation.^74^ Another useful isoquinoline alkaloid of *Berberis* sp. (Berberidaceae) namely berberine (**11**) (Figure 3), is used in various biological activities, especially in the field of cancer therapy. It has been studied that compound **11** inhibits the growth of A549 and H1299 cells through several different mechanisms including AP-2/hTERT, Raf/MEK/ERK, NF-кB/COX-2, P13K/Akt, HIF-1α/VEGF, and cytochrome c/caspase.^75^ Anoikis is known as a process, during which apoptotic cells are detached from the extracellular matrix and neighboring cells. Anoikis-resistant agents are found to develop in metastatic cancer cells, which primarily occur through survival and apoptotic mechanisms including ERK and Akt. Reniermycin M (**12**) (Figure 3), a bistetrahydroisoquinolinequinone alkaloid isolated from a marine blue sponge,* Xestospongia* sp., was sensitive to Anoikis-resistant H460 lung cancer cells through suppressing p-ERK, Akt, and total Akt and decreased levels of anti-apoptotic proteins including MCL1 and BCL2.^76^

####  Steroidal alkaloids

 Overexpression of oncogenic microRNA-21 in lung cancer cells resulted in the invasion of cancerous cells by targeting reversion-inducing cysteine-rich protein with kazal motifs (RECK). Solasodine (**13**) (Figure 3) is a terpenic aglycone of solanine alkaloid found in eggplant, which inhibited lung cancer cell invasion. Compound **13** potentially down-regulated miR-21 expression and elevated RECK in A549 through the suppression of the P13K/Akt pathway.^77^

####  Carbazole alkaloids

 Mahanine (**14**) (Figure 3), a carbazole alkaloid of *Murraya koenigii* (L.) Sprengel (Rutaceae), defected the gene expression of rapamycin component of m-TORC2 (rictor) in A549 and H1299 cells, and the consequent inhibition of rictor expression caused the reduction of p-Akt and p-mTOR levels.^78^

####  Miscellaneous alkaloids

 Wang et al studied the effects of Piperlongumine (**15**) (Figure 3), found in long pepper, against A549 and docetaxel-resistant A549 (A549/DTX) cells. Compound **15** not only induced apoptosis in both cell lines through modulating the P13K/Akt/mTOR^79^ but also exhibited an anti-proliferative activity in A549 cells by multiple processes, such as decreased expression of CDK6, CDK4, and cyclin D1, declined generation of ROS, inhibition of Akt phosphorylation, and NF-кB inactivation.^80^ Several kinds of cancers, including NSCLC, are associated with the overexpression of the cancerous inhibitor of protein phosphatase 2A (CIP2A) oncoprotein. CIP2A regulates c-Myc stability through inactivating protein phosphatase 2A (PPA2) and causes pAkt activity. Ethoxysanguinarine (**16**) (Figure 3) is a benzophenanthridine alkaloid found in Papaveraceae plants such as *Macleaya cordata *(Willd) R. Br. A study on the effects of **16** towards H1975 and A549 cells showed a down-regulation of CIP2A and CIP2A downstream molecules, pAkt and c-Myc, and activated PP2A. In addition, combining **16** with cisplatin increased apoptosis in lung cancer cell lines.^71^ Tumor necrosis factor α (TNF-α) promotes the survival and metastasis progressions in lung cancer; therefore, identifying the natural compounds with the ability to suppress TNF-α-induced survival signaling is viable in cancer therapy. Dicentrine (**17**) (Figure 3), an aporphin alkaloid in various medicinal plants such as *Lindera megaphylla*Hemsl., significantly enhanced TNF-α-mediated apoptosis in A549 cells through the cleavage of the caspase family and PARP and reduced the expression of anti-apoptotic proteins including cIAP2, c-FLIP, and Bcl-xl. It could also decrease the expression enhancement of TNF-α-induced metastasis-associated proteins and inhibited TNF-α-induced AP-1 and NF-кB activation. In addition, compound **17** blocked the phosphorylation of TNF-α-activated ERK1/2 and Akt signaling pathways.^81^ Leonurine hydrochloride (**18**) (Figure 3), the major alkaloid of *Leonurus japonicus* Houtt. (Lamiaceae), induced apoptosis in H292 cells by the mitochondrial-dependent pathway associated with ROS production and loss of MMP. In addition, it reduced the phosphorylated Akt level.^79^

###  Flavonoids regulate the PI3K/Akt/mTOR signaling in lung cancer

**Figure 4:**
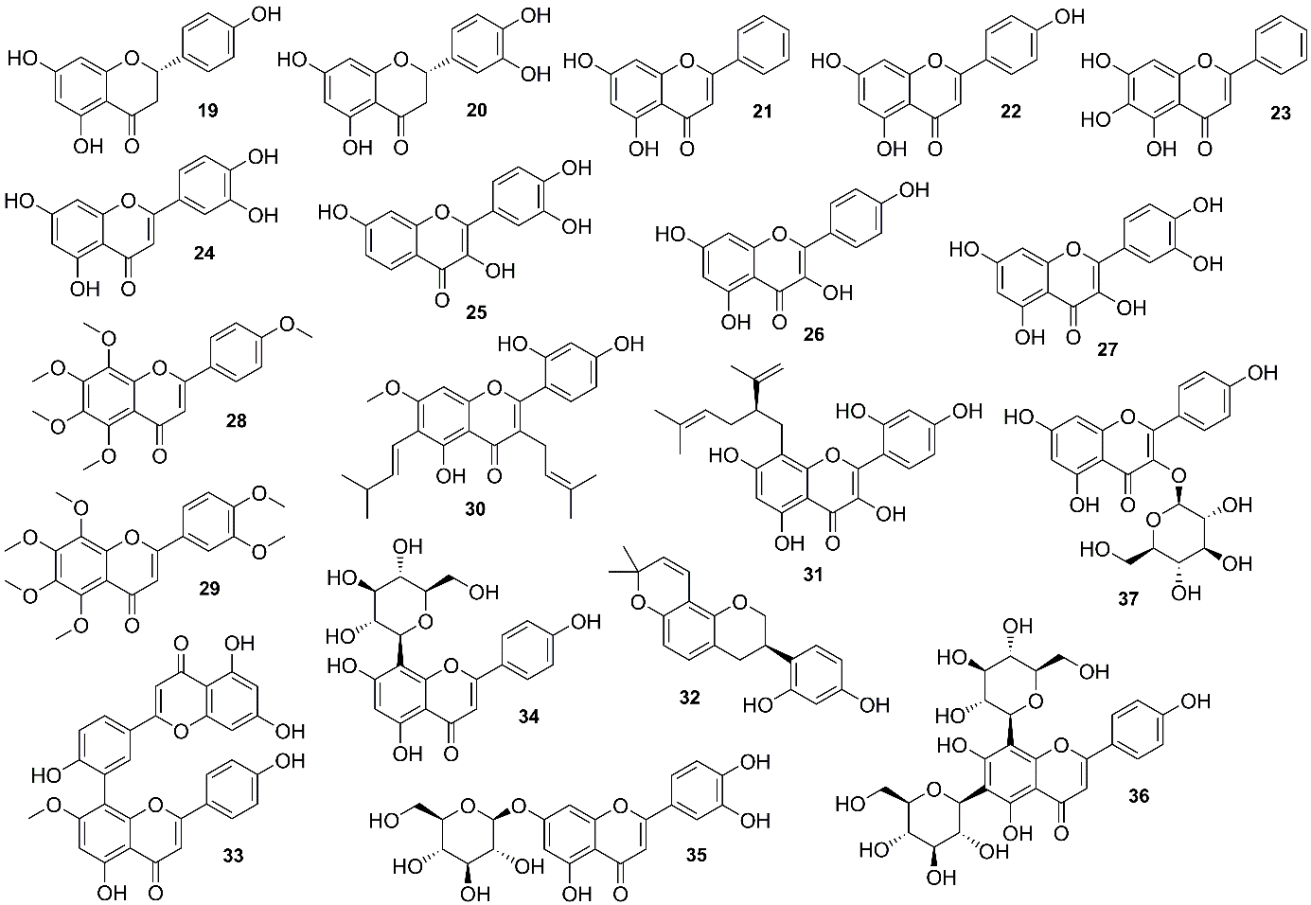
Chemical structures of natural flavonoids (19-37)

 To have a comprehensive literature review, the anti-cancer natural flavonoids targeting the PI3K/Akt/mTOR signaling pathway in lung cancer are categorized according to their chemical classes (Figure 4) while the mechanisms of actions are also summarized for each class of flavonoids where available (Table S2; Supplementary file 1).

####  Flavanones

 An experimental study on a *Citrus* flavonoid, naringenin (**19**) (Figure 4), revealed an anti-migration activity in A549 cells, which was associated with reducing MMP-9 and MMP-2 activities and inhibition of Akt activity in a dose-dependent manner.^82,83^ Eriodictyol (a bitter-masking flavanone; **20**) is a common flavonoid of the plant kingdom. Zhang et al showed that compound **20** (Figure 4) constructed A549 cell death by inducing the G2/M cell cycle arrest, mitochondrial apoptosis, and the mTOR/PI3K/Akt pathway suppression.^84^

####  Flavones

 Chrysin (**21**) (Figure 4), a widely distributed flavone in medicinal plants, promoted A549 cells’ apoptosis and growth inhibition of Akt/mTOR contributed by AMPK activation in A549 cells.^85^ Apigenin (**22**) (Figure 4), another common flavone found in vegetables and fruits, has been considered as a potential agent with anti-invasion, anti-migration, and anti-proliferation activities in A549 cells. It was indicated that apigenin inhibited phosphorylation and activation of Akt as well as the gene expression of Akt downstream including MMP-9, GSK-3β, and HEF1.^86^ In addition, combined apigenin and TRAIL up-regulated death receptors 4 and 5 levels in a p53-dependent manner resulted in apoptosis in NSCLC cells. This combined product could also suppress ERK activation and translocation of NF-кB and PI3K/Akt pathways.^87^ Compound **22** could suppress the invasion and migration of lung cancer cells with different EGFRs via inhibiting Snail/Slug-mediated EMT attributed to its capacity in Akt inactivation.^88^ Inhibition of epithelial-mesenchymal transition (EMT) and induction of apoptosis via PI3K/Akt/NF-кB involved two mechanisms induced by baicalein (**23**) (Figure 4), a bioactive flavone of *Scutellaria*species (Lamiaceae), to overcome the resistance of lung adenocarcinoma cells to cisplatin.^89^ EMT is a key process in promoting cancer progression, metastasis, and invasion, promoted through TGF-β, a multifunctional cytokine overexpressed in many types of cancers. One study showed that luteolin (**24**) (Figure 4), a common dietary flavone, acted as anti-adhesive molecules, and E-cadherin attenuated the activation of the PI3K–Akt–IκBa–NF-κB–Snail pathway.^90^

####  Flavonols

 Fisetin (**25**) (Figure 4) is a dietary flavonol, which has shown a selective inhibition of mTOR signaling and PI3K/Akt in NSCLC cells without side effects on normal human bronchial epithelial cells.^91^ Likewise, effects of kaempferol (**26**) (Figure 4) were evaluated on A549 cells by Han et al. The authors claimed that compound **26** inhibited proliferation and enhanced autophagy and apoptosis through the PTEN/PI3K/Akt pathway; however, another study reported that the inactivation of MEK/ERK was necessary for kaempferol-induced apoptosis in lung cancer cells.^92,93^ Another abundant bioflavonoid, quercetin (**27**) (Figure 4), was claimed to be an anti-metastatic agent on NSCLC cells through Snail-dependent Akt activity up-regulated by maspin and Snail-independent ADAM9 pathways.^94^ Inhibition of Akt was also involved in sensitizing TRAIL-induced cytotoxicity in NSCLC by **27**.^95^ A study carried out by Nguyen et al revealed quercetin-induced apoptosis in A549 cells by inactivating MEK/ERK and Akt-1 and influencing the expression of the Bcl-2 family of proteins.^96^ In addition, quercetin-metabolite-enriched plasma obtained from Mongolian gerbils fed with quercetin for 24 hours (100 mg/kg body/wt/week) was shown to reduce the growth of A549 cells and increase PARP-γ expression, which was associated with reduced phosphorylation of Akt.^97^

####  Polymethoxy flavones

 Polymethoxylated flavone of *Citrus* fruits peel, tangeretin (**28**) (Figure 4), suppressed COX-2 expression induced by IL-1β in A549 cells. This effect might be mediated through inhibiting the phosphorylation of JNK, p38 MAPK, and AKT as well as blocking NF-кB translocation.^98^ Another *Citrus* polymethoxy flavone, nobiletin (**29**) (Figure 4), revealed an ability to improve Adriamycin (ADR) resistance of A549/ADR cells. It might also increase ADR accumulation by inhibiting the MRP1 expression via down-regulating the expression of β-catenin, GSK-3β, Akt, and MYCN.^83^

####  Isoprenylated flavonoids

 Artocarpin (**30**) (Figure 4), a major flavonoid of *Artocarpus* species (Moraceae), has been observed to induce apoptosis through Nox2/p47^phox^ activation and enhance ROS generation, which caused PI3K/Akt^s473^/p53-independent activation of the NF-кB/c-Myc/Noxa pathway in H1299 and A549 cells.^99^ One isolated flavonoid of *Sophora flavescens* Aiton (Fabaceae), kushenol z (**31**) (Figure 4), demonstrated an anti-proliferative effect by inhibiting the cAMP-PDE pathway and, subsequently, increasing PKA activity and led to the mTOR pathway inhibition in A549 cells. Additionally, down-regulation of Akt was involved in the inhibition of cell proliferation by **31**.^100^

####  Prenylated isoflavanes

 Glabridin (**32**) (Figure 4), the flavonoid compound of licorice (*Glycyrrhiza glabra* L.; Fabaceae), has been reported to have anti-metastasis, anti-migration, and anti-invasion effects on A549 cells, and cooperation of FAK/Src with Akt was considered to have a key role in glabridin-mediated cell migration.^101^

####  Biflavones

 Wang et al evaluated the anti-metastasis and anti-invasion activity of sotetsuflavone (**33**) (Figure 4), an isolated flavonoid of *Cycas revoluta *Thunb. (Cycadaceae), in A549 cells, which occurred by reversing EMT and inhibiting angiogenesis. Compound **33** suppressed TNF-α/NF-кB and PI3K/Akt pathways involved in the down-regulation of HIF-1α with a key role in the anti-transfer activity of **33**.^102^

####  Flavone and flavonol glycosides

 In an experimental study, Liu et alshowed that vitexin (**34**) (Figure 4), a flavonoid of *Crataegus pinnatifida *Bunge (Rosaceae), induced apoptosis in A549 cells, partly through PI3K/Akt/mTOR signaling.^103^ Luteoloside (Figure 4) (also known as cynaroside; **35**), found in *Gentiana macrophylla *Pall. (Gentianaceae), induced autophagy in A549 cells by inhibiting p-p70S6K, p-mTOR, and p-Akt, which were correlated with ROS generation.^104^ A flavonoid glycoside extracted from *Dendrobium officinale* Kimura et Migo (Orchidaceae), vicenin II (Figure 4) (vitexin 8-C-glycosyl flavone; **36**), inhibited metastasis by suppressing EMT activated by TGF-β1 as the promotion of EMT in A549 cells. Deactivation of PI3K/Akt/mTOR and TGF-β/SMAD pathways was involved in this anti-metastatic activity.^105,106^

 One of the mechanisms involved in the induced apoptosis of lung cancer cells by astragaline (**37**) (Figure 4), a flavonoid of *Rosa agrestis* Savi (Rosaceae), is the inhibition of PI3K and Akt phosphorylation, which could inhibit IкBα degradation, p56 translocation, and Bcl-xl and Bcl-2 expression.^107^

## Feature prospective and conclusions

 The Akt/mTOR signaling pathway plays an essential role in cell survival, growth, and proliferation. During cancer cell development and tumorigenesis, the up-regulation of Akt/mTOR signaling occurs by either carcinogens or several genetic mutations of upstream regulators. In addition, this pathway could be considered as the main core of all signaling pathways in cancer cells. Associated with various aspects of cancer cells, the Akt/mTOR pathway also includes the malignant progression and radio/chemotherapy resistance in patients with lung cancer. Therefore, targeting the pivotal pathway is one of the most efficient strategies for cancer therapy that can guide researchers to select the potential natural metabolites for further investigations. Numerous clinically approved inhibitors of the Akt/mTOR signaling components are capable of regulating this pathway; however, they are connected with unwanted side effects. Down-regulation of the Akt/mTOR pathway in the early stages of tumor growth could lead to tumor growth inhibition. Targeting each of the upstream proteins of Akt can inhibit or reduce the activity of the Akt/mTOR pathway. Herbal medicines along with their preparations are attractive and increasingly popular in various health care systems. Natural metabolites are the most potential agents because of their eminent efficacy and safety. To the best of our knowledge, we have summarized some classes of natural products, such as alkaloids and flavonoids, possessing anticancer properties through the various mechanisms, especially targeting Akt/mTOR signaling. The most significant flavonoids discussed in the present study showed an Akt regulation in various kinds of lung cancer cells. In addition to the effective activity of aglycone, the activity of flavonoids might be related to their structural features bearing *ortho* hydroxy groups in the B ring along with meta hydroxy groups in the A ring as well as the carbonyl group in ring C (C-4).^108^ On the other hand, the backbone of quinolizidine, indole, and isoquinoline alkaloids could be selected as promising compounds for further studies. Taken together, naturally occurring metabolites are now used for developing alternative therapeutic strategies as they increase the action of traditional treatment methods through their multi-targeting capacity. Some studies revealed that the secondary metabolites showed stronger effects against cancer cells in the presence of cisplatin. Hence, a smart combination approach can target the PI3K/AKT/mTOR pathway in lung cancer cells. However, there are significant challenges associated with bioactive the PI3-kinase/Akt/mTOR inhibitors, which have limited their uses in cancer chemoprevention development. For instance, problems associated with flavonoids included their poor extraction yield, complicated purification methods, and pharmacokinetic/pharmacodynamic properties such as bioavailability, drug–drug interactions, and metabolic instability, which can be improved by effective drug delivery systems.^109^ In the case of alkaloids, inappropriate water solubility and low bioavailability are major challenges limiting their uses in oral administration. Nevertheless, water solubility and efficacy can increase by using semisynthetic and biochemical transformation approaches.^13^

 Overall, the PI3K/Akt/mTOR regulators are of more interest to researchers to unravel the real power of the Akt/mTOR targeting strategy in lung cancer therapy. In the next steps, clinical trials using natural compounds targeting Akt/mTOR in combination with standard treatments are vigorously suggested for future studies.

## Acknowledgments

 The authors with to thank University of Tehran for logistical supports.

## Author Contributions

**Conceptualization:** Sommayeh Ghareghomi, Shahin Ahmadian.

**Investigation:** Sommayeh Ghareghomi, Vahideh Atabaki, Naseh Abdollahzadeh.

**Project administration:** Shahin Ahmadian, Salar Hafez Ghoran.

**Resources: **Sommayeh Ghareghomi, Vahideh Atabaki, Salar Hafez Ghoran.

**Software:** Salar Hafez Ghoran.

**Supervision:** Shahin Ahmadian, Salar Hafez Ghoran

**Validation: **Shahin Ahmadian, Salar Hafez Ghoran

**Visualization:** Sommayeh Ghareghomi, Vahideh Atabaki, Salar Hafez Ghoran.

**Writing – original draft: **Sommayeh Ghareghomi, Vahideh Atabaki, Naseh Abdollahzadeh.

**Writing – review & editing: **Shahin Ahmadian, Salar Hafez Ghoran.

## Ethical Issues

 Not applicable.

## Conflict of Interests

 The authors declare no potential competing interest.

## Supplementary Files

Supplementary file 1 contains Tables S1 and S2.
