# Supplementary material for: Bioactive PI3-kinase/Akt/mTOR Inhibitors in Targeted Lung Cancer Therapy
Source: Adv Pharm Bull. 2021 Oct 9;13(1):24–35. doi: 10.34172/apb.2023.003 (PMC9871280; doi:10.34172/apb.2023.003)
Supplement: Supplementary file 1 — contains Tables S1 and S2. [file apb-13-24-s001.pdf]

Adv Pharm Bull, 2023, 13(1), 24-35

doi: 10.34172/apb.2023.003

https://apb.tbzmed.ac.ir

Bioactive PI3-kinase/Akt/mTOR Inhibitors in Targeted Lung Cancer Therapy

Somayyeh Ghareghomi[1], Vahideh Atabaki[2], Naseh Abdollahzadeh[3], Shahin Ahmadian[1*], Salar Hafez Ghoran[4,5*]

[1]Department of Biochemistry, Institute of Biochemistry and Biophysics (IBB), University of Tehran, Tehran, Iran.
[2]Department of Pharmacognosy and Pharmaceutical Biotechnology, Faculty of Pharmacy, Hormozgan University of Medical Sciences, Bandar Abbas, Iran.
[3]Neurophysiology Research Center, Cellular and Molecular Medicine Institute, Urmia University of Medical Sciences, Urmia, Iran.
[4]Phytochemistry Research Center, Shahid Beheshti University of Medical Sciences, Tehran, Iran.
[5]Medicinal Plants Research Center, Yasuj University of Medical Sciences, Yasuj, Iran.

Supplementary file 1

Bioactive PI3-kinase/Akt/mTOR Inhibitors in Targeted Lung Cancer Therapy

Somayyeh Ghareghomi[1], Vahideh Atabaki[2], Naseh Abdollahzadeh[3], Shahin Ahmadian[1]*, Salar Hafez Ghoran[4,5]*

[1]Department of Biochemistry, Institute of Biochemistry and Biophysics (IBB), University of Tehran, Tehran, Iran.
[2]Department of Pharmacognosy and Pharmaceutical Biotechnology, School of Pharmacy, Shahid Beheshti University of Medical Sciences, Tehran, Iran.
[3]Department of Physiology, School of Medicine, Tabriz University of Medical Sciences, Tabriz, Iran.
[4]Department of Chemistry, Faculty of Science, Golestan University, Gorgan, Iran.
[5]Medicinal Plants Research Center, Yasuj University of Medical Sciences, Yasuj, Iran.

Table S1. Summary of potential alkaloids targeting Akt/mTOR pathway in lung cancer cells.

Compd.	Mechanism of action	Assays	Ref.
Cytisine (**1**)	– Growth inhibition in cell lines including A549, NCIH23 and NCI-H460,<br>– Cell cycle arrest in G2/M phase in A549,<br>– Induction of mitochondrial apoptosis in A549 and NCI-H460 cancer cells,<br>– Increasing expression levels of p-38, p-JNK, BAD, cytochrome c, cleaved caspase and	– Cell counting kit-8 assay,<br>– Flow cytometry using Annexine V-FITC/propidium iodide staining,<br>– Western blotting, Measurement of ROS level with	[1]

	PARP, decreasing expression levels of Bcl-2, p-ERK, p-STAT3 and NF-κB (p65).	DCFH-DA,<br>– Measurement of tumor volume using formula 1/2(L×W2).	
Matrine (**2**)	– Reduction in cell viability of A549 and 95D cancer cell,<br>– Induction of early apoptotic pathway in dose and time-dependent manner,<br>– Inactivation of Akt in a dose-dependent manner.	– MTT assay,<br>– Flow cytometry,<br>– Western blotting.	[2]
Wasoxymatrine (**3**)	– Suppression of cell proliferation in A549, H1974 and HCC287 cancer cells,<br>– Decrease in EGRF phosphorylation and inactivation of ERK1/2 and Akt,<br>– Cell cycle arrest in G0/G1 phase,<br>– Tumor growth inhibition in HCC827 xenograft model	– MTS assay,<br>– Western blotting,<br>– Flow cytometry by using Annexine V-FITC/propidium iodide staining, Calculation of tumor volume using formula 1/2(L×W2)),<br>– Immunohistochemical analysis.	[3]
Evodiamine (**4**)	– Induction of arrest in G2/S phase, Inhibition of cell proliferation and Induction of apoptosis and ROS production in A549 cancer cells,<br>– Downregulation of mRNA expression levels of survivin, Bcl-2, cyclin B1, p-Src, SHH and GLI1,<br>– Reduction in telomerase activity, mRNA expression level of GLI1 and SHH and intracellular concentration of GSH,<br>– Decrease in NF-κB and Akt phosphorylation levels.	– MTS assay,<br>– Flow cytometry,<br>– Immunoblotting analysis,<br>– SYBR Green real-time PCR, Commercial GSH determination kit,<br>– Immunoblotting and Western blotting.	[4]
Hirsutine (**5**)	– Selective inhibition of cell proliferation and Induction caspase-dependent apoptosis in A549 cells,<br>– Decreasing mitochondrial membrane potential (ΔΨ) and depletion of ATP, Induction of release cytochrome c and ROS generation,<br>– Increasing association between cyclophilin-D (CypD) and adenine nucleotide translocator 1 (ANT1),<br>– Decrease in Rho-associated coiled-coil kinase (ROCK1) level and increase of p-PTEN levels,<br>– Inactivation of P13K/Akt pathway.	– Cell counting kit-8 assay,<br>– Flow cytometry by using Annexine V-FITC/propidium iodide staining, Confocal microscopy with JC-1 staining,<br>– ATP determination kit,<br>– Western blot analysis,<br>– DCF-DA staining, Immunoprecipitation assay.	[5]
Chaetoglobosin K (**6**)	– Inhibition of cell growth in H2009 and WB-ras cells<br>– Decrease in JNK and Akt phosphorylation<br>– Decrease in phosphorylation of active sites of c-JUN, ATF-2, MDM2 and Rac1	– Cell growth assay with counting cells per dish with hemocytometer, Western blotting.	[6]
Neferine (**7**)	– Reduction in cell viability of A549 cells,<br>– Acidic vesicular organelles (AVO) formation as the characteristic of autophagy,<br>– Down regulation of Akt/m-TOR pathway,<br>– Hyper-generation of ROS, depletion of GSH levels and lipid peroxidation (LPO) content,<br>– Increased p53, Bax, caspase-3 and caspase-9 expression and decrease in Bcl-2 gene expression,	– MTT assay,<br>– Acridine orange staining,<br>– Western blotting,<br>– Fluorometric assay,<br>– Light microscopy Real time-quantitative PCR analysis (RT-qPCR).	[7]<br>[8]

	– Decrease in gene expressions of PI3K/Akt/mTOR,<br>– Decrease in mRNA expressions of COX-2, NF-κB, VEGF and CYP2E1.		
Noscapine (**8**)	– Inhibition of cell proliferation in H460 and A549 cells,<br>– Reduction in tumor volume in nude mice bearing H460 xenograft lung tumor,<br>– Combination of noscapine and cisplatin induced apoptosis in H460, A549 cells and nude mice bearing H460 xenograft lung tumor,<br>– Increase in expression of p53, p21, caspase-3, cleaved caspase-3, cleaved PARP and Bax and decrease in expression of Akt, p-Akt, cyclin D1, survivin, Bcl-2 in H460 and A549 cells treated with combination of noscapine and cisplatin.	– Crystal violet dye assay,<br>– TUNEL assay,<br>– Western blotting.	[9]
Krukovine (**9**)	– Growth inhibition, Inhibition of cell colony formation, Induction of cell cycle arrest at G1 phase and apoptosis in H460 and A549 cancer cells,<br>– Inhibition of RAF/ERK pathway and Akt inactivation.	– MTT assay,<br>– Colony formation assay by staining with crystal violet,<br>– Flow cytometry and Western blotting.	[10]
Tetrandrine (**10**)	– Decreasing proliferation of A549 cancer cells and Induction of apoptosis,<br>– Decreasing Bcl-x, increasing Bid and Bax levels and Suppression of Akt and ERK phosphorylation.	– MTT assay,<br>– Flow cytometry by using Annexine V-FITC/propidium iodide staining,<br>– Western blotting.	[11]
Berberine (**11**)	– Inhibition of cell proliferation and migration and colony formation in A549 and H1299 cells,<br>– Increasing apoptotic cells in A549 and H1299 cells,<br>– Increasing expression levels of cleaved caspase3/7/9, cleaved PARP, Bax protein and decreasing Bcl-2 protein,<br>– Decrease in phosphorylated Akt and ERK1/2 levels, COX-2 expression,<br>– Inhibition of expression of AP-2α, AP-2β, hTERT, HIF-1α, VEGF, at mRNA and protein levels,<br>– Translocation of p50 and p65 NF-κB from cell nuclei to cytoplasm and Inhibition in binding of p50/p65 NF-κB to COX-2.	– Wound-healing assay,<br>– MTT assay,<br>– Anchorage independent colony formation assay,<br>– Annexin-V staining-based fluorescence-activated cell sorter,<br>– Western blotting, RT-PCR,<br>– Immunofluorescence assay,<br>– Streptavidin-agarose pulldown assay.	[12]
Reniermycin M (**12**)	– Growth inhibition of H460 cancer cells,<br>– Inhibition of aggregate formation in a dose-dependent manner,<br>– Decreasing p-Akt, p-ERK, BCL2 and MCL1 levels in a dose-dependent manner.	– Water-soluble tetrazolium salt assay,<br>– Light microscopy,<br>– Western blotting	[13]
Solasodine (**13**)	– Cytotoxic activity on A549 cancer cells<br>– Suppressing cell invasion in dose-dependent manner<br>– Suppression of mRNA expression of MMP-2, MMP-9 and extracellular inducer of matrix metalloproteinase (EMMPRIN)<br>– Elevation of expression of tissue inhibitor of metalloproteinase-1 (TIMP-1), TIMP-2 and reversion-inducing cysteine-rich protein with kazal motifs (RECK)<br>– Down-regulation of microRNA-21 expression and PI3K/Akt phosphorylation in dose-dependent manner.	– MTT assay,<br>– Boyden Chamber Invasion assay,<br>– RT-qPCR,<br>– SDS-PAGE and western blotting.	[14]
Mahanine (**14**)	– Significant growth inhibition in A549 and H1299 cells,	– MTT assay and cells counting with hemocytometer	[15]

	– Deactivation of Akt and reduction in m-TOR, PDK1 and Rictor level,<br>– Induction of apoptosis through suppression of Rictor.	after staining with trypan blue,<br>– Immunoblot and qPCR analysis,<br>– Annexine V-Cyc3 detection assay.	
Piperlongumine (**15**)	– Inhibition of growth, proliferation and colony formation in H549 and A549/DTX cancer cells,<br>– Induction of apoptosis and autophagy in H460 and A549/DTX cancer cells,<br>– Cleavage of PARP, reduction of Bcl-2 level,<br>– Induction of cell cycle arrest in S phase and ROS generation,<br>– Down regulation of cyclin D1, CDK4, CDK6 and Rb,<br>– Decreasing Akt phosphorylation and increasing ERK1/2 phosphorylation,<br>– NF-κB inactivation through Akt dephosphorylation.	– MTT assay,<br>– Colony formation assay by staining with hamatoxlin,<br>– Flow cytometry,<br>– Western blotting,<br>– TUNEL assay,<br>– Immunohistochemical assay.	[16]<br>[17]
Ethoxysanguinarine (**16**)	– Reduction in gene expression of CIP2A in A549 cells,<br>– Down regulation of CIP2A in H1975 and A549 cells through a time-dependent manner,<br>– Down regulation of p-Akt and p-Myc in H1975 and A549 cells<br>– Up regulation of PP2A,<br>– Cleavage of PARP, decrease in procaspase-8 and procaspase-9 in H1975 and A549 cell,<br>– Inhibition of cell proliferation and induction of apoptosis in H1975 and A549 cells.	– RT-qPCR,<br>– Western blotting, MTT assay,<br>– Flow cytometry by using Annexine V-FITC/propidium iodide staining.	[18]
Dicentrine (**17**)	– Reduction in cell viability, invasion and migration in A549 cancer cells,<br>– Increasing apoptotic cells<br>– Increasing cleavage of caspase-3, caspase-8, caspase-9, PARP and death-inducing signaling complex (DISC) formation<br>– Overexpression of c-IAP2, c-FLIP and Bcl-xl<br>– Inhibition of TNF-α induced expression of MT1-MMP, MMP-9, plasminogen activator receptor (Upar), intercellular adhesion molecule 1 (ICAM-1) and Cox-2,<br>– Inhibition of TNF-α induced NF-Kb, AP-1 activation and TNF-α-induced Akt phosphorylation.	– MTT assay,<br>– PI staining assay and flow cytometry analysis,<br>– Modified Boyden chamber,<br>– Western blotting.	[19]
Leonurine hydrochloride (**18**)	– Inhibition of cell proliferation and cell cycle arrest at G0/G1 phase in H292 cells,<br>– Induction apoptosis in H292 cells<br>– Increasing MAPK phosphorylation and decrease Akt phosphorylation,<br>– Increasing ROS accumulation and mRNA ratio of Bax/Bcl-2 and mRNA levels of caspase-3 and caspase-9.	– MTT assay,<br>– Flow cytometry,<br>– Western blotting,<br>– RT-q PCR	[20]

Table S2. Summary of potential flavonoids targeting Akt/mTOR pathway in lung cancer cells

Compd.	Mechanism of action	Assays	Ref.
Naringenin (**19**)	– Reduction of A549 cells migration,<br>– Inhibition of MMP-2 and MMP-9 activities,	– Wound healing,<br>– Boyden chamber assays,	[21]

	– Reduction in phosphorylation of the AKT/total AKT ratio.	– Gelatin zymography analysis,<br>– RT-PCR and Western blotting.	
Eriodictyol (**20**)	– Cytotoxic activity in A549 cells,<br>– Increased apoptotic cell death, DNA damage and cell cycle arrest in G2/M phase,<br>– Increasing Bax/Bcl-2 ratio,<br>– Downregulation of m-TOR, pm-TOR and PI3K/Akt protein expression levels.	– MTT assay,<br>– Annexin V/PI staining and flow cytometry,<br>– Comet assay,<br>– Muse Cell Analyzer and Muse Cell Cycle Kit,<br>– DOC6 and flow cytometry,<br>– Western blotting.	[22]
Chrysin (**21**)	– Growth inhibition and apoptosis by inhibition of Akt/mTOR activation,<br>– Promotion of A549 cell death and growth inhibition by increasing doxorubicin-induced AMPK activation.	– Western blotting,<br>– MTT assay and PI staining.	[23]
Apigenin (**22**)	– Decreasing cell viability, proliferation and migration in A549 cell,<br>– Decrease in Akt, p-Akt levels, MMP-2, MMP-9, GSK-3β, and HEF1 expression<br>– Downregulation of N-cadherin, and the EMT-promoting transcription factors,<br>– Suppression of Snail family mediated to AKT inactivation<br>– Suppression of clonogenic growth in A549 and H1299 cells,<br>– Cleavaging procaspase-8, procaspase-9, procaspase-3, and PARP in cells treated with combination of apigenin and TRAIL,<br>– Increase in levels of Bax and Bad and reduction in Bcl-2 and Bcl-xl levels in cells treated with combination of apigenin and TRAIL,<br>– Inhibition of NF-kB p65 nuclear translocation<br>– Decrease in c-FLIP expression in cells treated with combination of apigenin and TRAIL,<br>– Decrease in phosphorylation of AKT and PI3K after co-treatment of apigenin and TRAIL,<br>– Increasing phospho-JNK in cells co-treated with apigenin and TRAIL,<br>– Upregulating DR4 and DR5 levels, elevation of p53 level, activation of MAPK p38, and suppression of the MAPK ERK in cells co-treated with apigenin and TRAIL.	– MTT assay,<br>– Colony formation assay,<br>– Wound-healing assay,<br>– Transwell assay,<br>– Western blotting,<br>– Transwell migration and invasion assays,<br>– Colony-forming assays,<br>– Annexin V-FITC kit.	[24]<br>[25, 26]
Baicalein (**23**)	– Enhancement of diamminedichloroplatinum (CDDP) cytotoxicity and early apoptosis in CDDP-resistant A549 cells,<br>– Reduction in tumor growth of A549/CDDP xenograft model,<br>– Inhibition invasion and migration induced by CDDP,<br>– Blocking PI3K/Akt and NF-κB activity in A549/CDDP<br>– Decrease in anti-apoptotic genes including c-IAP1, c-IAP2, survivin and Bcl-Xl.	– MTT assay,<br>– Annexin V/PI staining,<br>– Weighting tumor size,<br>– Western blotting.	[27]
Luteolin (**24**)	– Blocking TGF-β1-induced EMT and EMT-related gene expression in A549 cells,<br>– Blocking TGF-β1-induced E-cadherin downregulation via Snail,<br>– Reverse of TGF-β1-induced decrease in IκBα,<br>– Inhibition of TGF-β1-induced phosphorylation of PI3K/Akt–mTOR pathway,<br>– Block of NF-κB binding to the Snail promoter region induced by TGF-β1.	– Trypan blue exclusion method,<br>– Annexin-V and PI staining,<br>– Western blot and RT-PCR analysis,<br>– Chromatin immunoprecipitation assay.	[28]
Fisetin (**25**)	– Decreased cell viability in A549 and H1792 cells,<br>– Interaction with mTOR complex in two sites and inhibition of downstream targets of mTOR including 4E-BP1, EIF4E and p70S6K,<br>– Activation of PTEN, as a tumor suppressor gene,<br>– Significant increase in phosphorylation of AMPKα,<br>– Inhibition of PI3K and Akt phosphorylation,<br>– Increase in TSC2 protein level at dose-dependent manner.	– MTT assay,<br>– Docking study,<br>– Immunoblot analysis,<br>– Western blotting and Phospho-Act ELISA kit,<br>– Immunoblot analysis and chemiluminescence detection.	[29]
Kaempferol (**26**)	– Reduction in cell viability in A549 cells and Induction of apoptosis,<br>– Decreasing Bcl-2 and Bcl-xL levels in a dose-dependent manner and p85 subunit of PI3K and total Akt,<br>– Increasing expression of Bad and Bax,<br>– Dose-dependent phosphorylation of MAPK, MEK1/2, c-Jun and cleaved PARP,<br>– Down-regulation of mRNA and protein levels of cyclinD1,	– MTT assay,<br>– TUNEL assay,<br>– Western blotting,<br>– Cell Counting Kit-8 (CCK-8) assay,<br>– RT-qPCR,<br>– Fluorescein isothiocynate (FITC)-conjugated annexin V and propidium iodide	[31]<br>[32]

	– Down-regulation of Bcl-2 and upregulation of Bax, cleaved caspase-3 and cleaved caspase-9,<br>– Decreasing phosphorylated levels of PI3K and AKT,<br>– Upregulation of miR-340 expression, mRNA and protein expression levels of PTEN.	[30].	
Quercetin (**27**)	– Suppression of migratory and invasive abilities in A549 and HCC827 cells,<br>– Induction of E-cadherin expression and suppression of N-cadherin and vimentin,<br>– Upregulation of p-AKT,<br>– Sensitization of TRAIL-induced cytotoxicity in H460, A549, H2009 and H1299 cells,<br>– Induction of apoptosis in H460 cells treated with TRAIL plus quercetin,<br>– Cleavaging PARP and activation of caspase-8 and -3 in cells treated with TRAIL plus quercetin,<br>– Inhibition of Akt phosphorylation and survivin in cells treated with TRAIL plus quercetin,<br>– Decrease in total Akt protein, phosphorylation of MEK1/2, ERK, JNK and c-Jun.	– MTT assay,<br>– Western blotting,<br>– Wound-closure,<br>– Transwell migration and Matrigel invasion assays,<br>– Immunofluorescence microscopy, LDH release assay, Staining with acridine orange-ethidium bromide and observation with microscope.	[33]<br>[34]<br>[35]
Tangeretin (**28**)	– Suppression of IL-1β induced COX-2 protein and m-RNA expression in A549 cells,<br>– Suppression of phosphorylation of ERK, p38 MAPK, JNK, and Akt induced by IL-1β in A549 cells,<br>– Inhibition of translocation of p65 NF-kB and degradation of IkBa induced by IL-1β,<br>– Inhibition of endogenous COX-2 expression in H1299 cells.	– Western blotting and RT-PCR.	[36]
Nobiletin (**29**)	– Decrease in cell viability in A549/ADR cells,<br>– Decrease in expression levels of MRP1 and neuroblastoma-derived MYC (MYCN),<br>– Decrease in GSK3β phosphorylation,<br>– Induction of apoptosis A549/ADR Cells and reduction in tumor volume,<br>– Cleavage of caspase-3 and c-PARP and the decrease in MRP1, survival and Bcl-xL.	– MTT assay,<br>– Western blotting,<br>– Confocal microscopy,<br>– Flow cytometry using PI.	[37]
Artocarpin (**30**)	– Proliferation inhibition of A549, H226 and H1299 cells,<br>– Induction of apoptosis and ROS generation,<br>– Induction of MAPK, ERK1/2 and Akt phosphorylation,<br>– Time-dependent promotion of phosphorylation of p53 and expression of PUMA, cytochrome c, Apaf-1 and cleaved caspase 3,<br>– NF-κB activation.	– MTT assay,<br>– ELISA assay,<br>– Western blotting,<br>– Annexin-V-FITC/PI,<br>– Immunocytochemistry & Receptor gene assay.	[38]
Kushenol z (**31**)	– Inhibition of NSCLS cells proliferation,<br>– Induction apoptotic body formation and apoptosis,<br>– Upregulating the ratio of Bax/Bcl-2 and cleavaging of caspase 3 and 9,<br>– Upregulated ERS marker, C/EBP Homologous Protein (CHOP),<br>– Increasing cleavage of caspase 7 and caspase12,<br>– Increasing the activity of PKA, inhibiting cAMP-PDE and Akt/mTOR pathway.	– Cell counting kit-8 (CCK-8) assay,<br>– Hoechst 33258 nuclear staining,<br>– Annexin V/PI staining,<br>– Western blotting.	[39]
Glabridin (**32**)	– Decrease in A549 cell migration and invasion,<br>– Increase in E-cadherin level and decrease in vimentin,<br>– Decrease in angiogenesis of A549 cells, expression of av and b3 integrin, phosphorylation of FAK, phosphorylation forms of AKT.	– Transwell migration and wound-healing assay,<br>– Immunoblot analysis,<br>– BD Bio-Coat Angiogenesis System, Immunoblot analysis,<br>– Immunoblot assay and Rho activation assay kit.	[40]
Sotetsuflavone (**33**)	– Dose-dependent inhibition of A549 cell migration and invasion,<br>– EMT reversing by upregulation of E-cadherin and downregulation of N-cadherin, vimentin and snail,<br>– Decreasing HIF-1α, NF-κB and TNF-α expression,<br>– Downregulation of MMP-9 and MMP-13 expression,<br>– Inhibition of PI3K/Akt pathway in time and dose-dependent manners.	– Scratch test and Transwell invasion assay,<br>– Western blotting, Immunoflufluorescence assay,<br>– PCR (RT-PCR)	[41]
Vitexin (**34**)	– Dose-dependent reduction in cell viability in A549 cells,<br>– Induction of apoptosis,<br>– Increasing loss of MMP,	– MTT assay,<br>– Flow cytometry analysis via Annexin-V/PI double staining,<br>– Weighting tumor volume,	[42]

	– Downregulation of Bcl2/Bax ratio and upregulation of cleavd-caspase-3,<br>– Reduction in p-Akt, p-P13K and p-mTOR levels.	– JC-1 staining,<br>– Western blotting.	
Luteoloside (**35**)	– Cytotoxic activity in A549 and H292 cells,<br>– Cell cycle arrest in G0/G1 phase and induction of autophagy,<br>– Upregulation of LC3-II and Beclin-1 expression and decrease of p62 and p-mTOR,<br>– Decrease in CyclinD1, CDK4 and Cyclin E,<br>– Downregulation of Erk1/2, inhibition of p-AKT, p-P70S6K and p-mTOR.	– MTT assay,<br>– Flow cytometry,<br>– MDC as florescent probe,<br>– Western blotting,<br>– H2DCFDA prob and flow cytometry.	[43]
Vicenin II (**36**)	– Reduction in cell viability of H23 cells both alone and in combination with radiation,<br>– Increasing ROS generation in combination with radiation,<br>– Increasing PI3KCA protein and mRNA levels in combination with radiation,<br>– Lowering levels of pAkt, Akt, and Akt1 mRNA in combination with radiation,<br>– Lowering levels of Bcl-2 protein and Bcl-2, Bcl-xL mRNA levels in combination with radiation.	– MTT assay and colony forming assay,<br>– Protein carbonyl assay by using 2,4 dinitrophenylhydrazine (DNPH),<br>– DCFH-DA,<br>– Western blotting,<br>– Quantitative real-time PCR,<br>– Ultra-structural study.	[44]
Astragaline (**37**)	– Selective cytotoxicity in A549 and H1299 cells,<br>– Induction of apoptosis in A549 and H1299 cells,<br>– Inhibition of the clonogenic growth of H1299 and A549 cells,<br>– Cleavaging of caspase-8, -9, -3, and PARP,<br>– Decreasing the expression of Bcl-xl and Bcl-2 and increasing in the expression of Bad and Bax,<br>– Decrease in p38 and ERK phosphorylation in a dose-dependent manner,<br>– Inhibition of the nucleus translocation of NF-κB.	– MTT assay,<br>– Propidium iodide staining [30],<br>– Colony-forming assay,<br>– Western blotting.	[45]

References

1. Xu WT, Li TZ, Li SM, Wang C, Wang H, Luo YH, et al. Cytisine exerts anti-tumour effects on lung cancer cells by modulating reactive oxygen species-mediated signalling pathways. *Artif Cells Nanomed Biotechnol* 2020;48(1):84-95. Doi:10.1080/21691401.2019.1699813
2. Niu H, Zhang Y, Wu B, Zhang Y, Jiang H, He P. Matrine induces the apoptosis of lung cancer cells through downregulation of inhibitor of apoptosis proteins and the Akt signaling pathway. *Oncol Rep* 2014;32(3):1087-1093. Doi:10.3892/or.2014.3273
3. Li W, Yu X, Tan S, Liu W, Zhou L, Liu H. Oxymatrine inhibits non–small cell lung cancer via suppression of EGFR signaling pathway. *Cancer Med* 2018;7(1):208-218. Doi:10.1002/cam4.1269
4. Mohan V, Agarwal R, Singh RP. A novel alkaloid, evodiamine causes nuclear localization of cytochrome-c and induces apoptosis independent of p53 in human lung cancer cells. *Biochem Biophys Res Commun* 2016;477(4):1065-1071. Doi:10.1016/j.bbrc.2016.07.037
5. Zhang R, Li G, Zhang Q, Tang Q, Huang J, Hu C, et al. Hirsutine induces mPTP-dependent apoptosis through ROCK1/PTEN/PI3K/GSK3β pathway in human lung cancer cells article. *Cell Death Dis* 2018;9(6). Doi:10.1038/s41419-018-0641-7
6. Ali A, Sidorova TS, Matesic DF. Dual modulation of JNK and Akt signaling pathways by chaetoglobosin K in human lung carcinoma and ras-transformed epithelial cells. *Invest New Drugs* 2013;31(3):525-534. Doi:10.1007/s10637-012-9883-x
7. Poornima P, Weng CF, Padma VV. Neferine from Nelumbo nucifera induces autophagy through the inhibition of PI3K/Akt/mTOR pathway and ROS hyper generation in A549 cells. *Food Chem* 2013;141(4):3598-3605. Doi:10.1016/j.foodchem.2013.05.138
8. Selvi SK, Vinoth A, Varadharajan T, Weng CF, Padma VV. Neferine augments therapeutic efficacy of cisplatin through ROS-mediated non-canonical autophagy in human lung adenocarcinoma (A549 cells). *Food Chem Toxicol* 2017;103:28-40. Doi:10.1016/j.fct.2017.02.020
9. Chougule M, Patel AR, Sachdeva P, Jackson T, Singh M. Anticancer activity of Noscapine, an opioid alkaloid in combination with Cisplatin in human non-small cell lung cancer. *Lung Cancer* 2011;71(3):271-282. Doi:10.1016/j.lungcan.2010.06.002
10. Lai H, Wang Y, Duan F, Li Y, Jiang Z, Luo L, et al. Krukovine suppresses KRAS-mutated lung Cancer cell growth and proliferation by inhibiting the RAF-ERK pathway and inactivating AKT pathway. *Front Pharmacol* 2018;9:958. Doi:10.3389/fphar.2018.00958
11. Cho HS, Chang SH, Chung YS, Shin JY, Park SJ, Lee ES, et al. Synergistic effect of ERK inhibition on tetrandrine-induced apoptosis in A549 human lung carcinoma cells. *J Vet Sci* 2009;10(1):23-28. Doi:10.4142/jvs.2009.10.1.23
12. Fu L, Chen W, Guo W, Wang J, Tian Y, Shi D, et al. Berberine Targets AP-2/hTERT, NF-κB/COX-2, HIF-1α/VEGF and Cytochrome-c/Caspase Signaling to Suppress Human Cancer Cell Growth. *PLoS one* 2013;8(7). Doi:10.1371/journal.pone.0069240
13. Sirimangkalakitti N, Chamni S, Suwanborirux K, Chanvorachote P. Renieramycin M sensitizes anoikis-resistant H460 lung cancer cells to anoikis. *Anticancer Res* 2016;36(4):1665-1671. PMID:27069144
14. Shen KH, Hung JH, Chang CW, Weng YT, Wu MJ, Chen PS. Solasodine inhibits invasion of human lung cancer cell through downregulation of miR-21 and MMPs expression. *Chem Biol Interact* 2017;268:129-135. Doi:10.1016/j.cbi.2017.03.005
15. Chatterjee P, Seal S, Mukherjee S, Kundu R, Bhuyan M, Barua NC, et al. A carbazole alkaloid deactivates mTOR through the suppression of rictor and that induces apoptosis in lung cancer cells. *Mol Cell Biochem* 2015;405(1-2):149-158. Doi:10.1007/s11010-015-2406-2
16. Wang F, Mao Y, You Q, Hua D, Cai D. Piperlongumine induces apoptosis and autophagy in human lung cancer cells through inhibition of PI3K/Akt/mTOR pathway. *Int J Immunopathol Pharmacol* 2015;28(3):362-373. Doi:10.1177/0394632015598849
17. Seok JS, Jeong CH, Petriello MC, Seo HG, Yoo H, Hong K, et al. Piperlongumine decreases cell proliferation and the expression of cell cycle-associated proteins by inhibiting Akt pathway in human lung cancer cells. *Food Chem Toxicol* 2018;111:9-18. Doi:10.1016/j.fct.2017.10.058
18. Liu Z, Ma L, Wen Z-S, Cheng Y-X, Zhou G-B. Ethoxysanguinarine induces inhibitory effects and downregulates CIP2A in lung cancer cells. *ACS Med Chem Lett* 2014;5(2):113-118. Doi:10.1021/ml400341k

19. Ooppachai C, Limtrakul P, Yodkeeree S. Dicentrine potentiates TNF-α-induced apoptosis and suppresses invasion of a549 lung adenocarcinoma cells via modulation of nf-κb and ap-1 activation. *Molecules* 2019;24(22):4100. Doi:10.3390/molecules24224100

20. Wang F, Mao Y, You Q, Hua D, Cai D. Piperlongumine induces apoptosis and autophagy in human lung cancer cells through inhibition of PI3K/Akt/mTOR pathway. *Int J Immunopathol Pharmacol* 2015;28(3):362-373. Doi:10.1177/0394632015598849

21. Chang HL, Chang YM, Lai SC, Chen KM, Wang KC, Chiu TT, et al. Naringenin inhibits migration of lung cancer cells via the inhibition of matrix metalloproteinases-2 and -9. *Exp Ther Med* 2017;13(2):739-744. Doi:10.3892/etm.2016.3994

22. Zhang Y, Zhang R, Ni H. Eriodictyol exerts potent anticancer activity against A549 human lung cancer cell line by inducing mitochondrial-mediated apoptosis, G2/M cell cycle arrest and inhibition of m-TOR/PI3K/Akt signalling pathway. *Arch Med Sci* 2020;16(2):446. Doi:10.5114/aoms.2019.85152

23. Shao J-j, Zhang A-p, Qin W, Zheng L, Zhu Y-f, Chen X. AMP-activated protein kinase (AMPK) activation is involved in chrysin-induced growth inhibition and apoptosis in cultured A549 lung cancer cells. *Biochem Biophys Res Commun* 2012;423(3):448-453. Doi:10.1016/j.bbrc.2012.05.123

24. Zhou Z, Tang M, Liu Y, Zhang Z, Lu R, Lu J. Apigenin inhibits cell proliferation, migration, and invasion by targeting Akt in the A549 human lung cancer cell line. *Anticancer Drugs* 2017;28(4):446-456. Doi:10.1097/CAD.0000000000000479

25. Chen M, Wang X, Zha D, Cai F, Zhang W, He Y, et al. Apigenin potentiates TRAIL therapy of non-small cell lung cancer via upregulating DR4/DR5 expression in a p53-dependent manner. *Sci Rep* 2016;6:35468. Doi:10.1038/srep35468

26. Chang J-H, Cheng C-W, Yang Y-C, Chen W-S, Hung W-Y, Chow J-M, et al. Downregulating CD26/DPPIV by apigenin modulates the interplay between Akt and Snail/Slug signaling to restrain metastasis of lung cancer with multiple EGFR statuses. *J Exper Clin Cancer Res* 2018;37(1):1-16. Doi:10.1186/s13046-018-0869-1

27. Yu M, Qi B, Xiaoxiang W, Xu J, Liu X. Baicalein increases cisplatin sensitivity of A549 lung adenocarcinoma cells via PI3K/Akt/NF-κB pathway. *Biomed Pharmacother* 2017;90:677-685. Doi:10.1016/j.biopha.2017.04.001

28. Chen K-C, Chen C-Y, Lin C-J, Yang T-Y, Chen T-H, Wu L-C, et al. Luteolin attenuates TGF-β1-induced epithelial–mesenchymal transition of lung cancer cells by interfering in the PI3K/Akt–NF-κB–Snail pathway. *Life Sci* 2013;93(24):924-933. Doi:10.1016/j.lfs.2013.10.004

29. Khan N, Afaq F, Khusro FH, Mustafa Adhami V, Suh Y, Mukhtar H. Dual inhibition of phosphatidylinositol 3-kinase/Akt and mammalian target of rapamycin signaling in human nonsmall cell lung cancer cells by a dietary flavonoid fisetin. *Int J Cancer* 2012;130(7):1695-1705. Doi:10.1002/ijc.26178

30. George VC, Dellaire G, Rupasinghe HV. Plant flavonoids in cancer chemoprevention: role in genome stability. *J Nutr Biochem* **2017;**45:1-14. Doi:10.1016/j.jnutbio.2016.11.007

31. Nguyen T, Tran E, Ong C, Lee S, Do P, Huynh T, et al. Kaempferol-induced growth inhibition and apoptosis in A549 lung cancer cells is mediated by activation of MEK-MAPK. *J Cell Physiol* 2003;197(1):110-121. Doi:10.1002/jcp.10340

32. Han X, Liu C-F, Gao N, Zhao J, Xu J. Kaempferol suppresses proliferation but increases apoptosis and autophagy by up-regulating microRNA-340 in human lung cancer cells. *Biomed Pharmacother* 2018;108:809-816. Doi:10.1016/j.biopha.2018.09.087

33. Chang J-H, Lai S-L, Chen W-S, Hung W-Y, Chow J-M, Hsiao M, et al. Quercetin suppresses the metastatic ability of lung cancer through inhibiting Snail-dependent Akt activation and Snail-independent ADAM9 expression pathways. *Biochim Biophys Acta* 2017;1864(10):1746-1758. Doi:10.1016/j.bbamcr.2017.06.017

34. Chen W, Wang X, Zhuang J, Zhang L, Lin Y. Induction of death receptor 5 and suppression of survivin contribute to sensitization of TRAIL-induced cytotoxicity by quercetin in non-small cell lung cancer cells. *Carcinogenesis* 2007;28(10):2114-2121. Doi:10.1093/carcin/bgm133

35. Nguyen T, Tran E, Nguyen T, Do P, Huynh T, Huynh H. The role of activated MEK-ERK pathway in quercetin-induced growth inhibition and apoptosis in A549 lung cancer cells. *Carcinogenesis* 2004;25(5):647-659. Doi:10.1093/carcin/bgh052

36. Chen K-H, Weng M-S, Lin J -K. Tangeretin suppresses IL-1β-induced cyclooxygenase (COX)-2 expression through inhibition of p38 MAPK, JNK, and AKT activation in human lung carcinoma cells. *Biochem Pharmacol* 2007;73(2):215-227. Doi:10.1016/j.bcp.2006.09.018

37. Moon JY, Manh Hung LV, Unno T, Cho SK. Nobiletin enhances chemosensitivity to adriamycin through modulation of the Akt/GSK3β/β–catenin/MYCN/MRP1 signaling pathway in A549 human non-small-cell lung cancer cells. *Nutrients* 2018;10(12):1829. Doi:10.3390/nu10121829

38. Tsai M-H, Liu J-F, Chiang Y-C, Hu SC-S, Hsu L-F, Lin Y-C, et al. Artocarpin, an isoprenyl flavonoid, induces p53-dependent or independent apoptosis via ROS-mediated MAPKs and Akt activation in non-small cell lung cancer cells. *Oncotarget* 2017;8(17):28342. Doi:10.18632/oncotarget.16058

39. Chen H, Yang J, Hao J, Lv Y, Chen L, Lin Q, et al. A novel flavonoid kushenol Z from *Sophora flavescens* mediates mTOR pathway by inhibiting phosphodiesterase and Akt activity to induce apoptosis in non-small-cell lung cancer cells. *Molecules* 2019;24(24):4425. Doi:10.3390/molecules24244425

40. Tsai Y-M, Yang C-J, Hsu Y-L, Wu L-Y, Tsai Y-C, Hung J-Y, et al. Glabridin inhibits migration, invasion, and angiogenesis of human non–small cell lung cancer A549 cells by inhibiting the FAK/Rho signaling pathway. *Integr Cancer Ther* 2011;10(4):341-349. Doi:10.1177/1534735410384860

41. Wang S, Yan Y, Cheng Z, Hu Y, Liu T. Sotetsuflavone suppresses invasion and metastasis in non-small-cell lung cancer A549 cells by reversing EMT via the TNF-α/NF-κB and PI3K/AKT signaling pathway. *Cell Death Discov* 2018;4(1):1-11. Doi:10.1038/s41420-018-0026-9

42. Liu X, Jiang Q, Liu H, Luo S. Vitexin induces apoptosis through mitochondrial pathway and PI3K/Akt/mTOR signaling in human non-small cell lung cancer A549 cells. *Biol Res* 2019;52(1):7. Doi:10.1186/s40659-019-0214-y

43. Zhou M, Shen S, Zhao X, Gong X. Luteoloside induces G0/G1 arrest and pro-death autophagy through the ROS-mediated AKT/mTOR/p70S6K signalling pathway in human non-small cell lung cancer cell lines. *Biochem Biophys Res Commun* 2017;494(1-2):263-269. Doi:10.1016/j.bbrc.2017.10.042

44. Baruah TJ, Kma L. Vicenin-2 acts as a radiosensitizer of the non-small cell lung cancer by lowering Akt expression. *BioFactors* 2019;45(2):200-210. Doi:10.1002/biof.1472

45. Chen M, Cai F, Zha D, Wang X, Zhang W, He Y, et al. Astragalin-induced cell death is caspase-dependent and enhances the susceptibility of lung cancer cells to tumor necrosis factor by inhibiting the NF-κB pathway. *Oncotarget* 2017;8(16):26941. Doi:10.18632/oncotarget.15264
